# Expression and significance of 90K/Mac-2BP in prostate cancer

**DOI:** 10.3892/etm.2012.768

**Published:** 2012-10-25

**Authors:** JIANXIN HU, JIAN HE, YOULIN KUANG, ZHENXING WANG, ZHAOLIN SUN, HENGCHENG ZHU, XIUHENG LIU

**Affiliations:** 1 Department of Urology, Guizhou People’s Hospital, Guiyang 550002;; 2 Department of Urology, The People’s Hospital of Wuhan University, Wuchang 430060, P.R. China

**Keywords:** prostate cancer, 90K, Mac-2BP, 2-DE patterns proteomics

## Abstract

The aim of this study was to detect the differences in 90K/Mac-2BP expression in prostate cancer, benign prostatic hyperplasia and normal prostate tissues, as well as to study the significance of 90K/Mac-2BP in the early diagnosis and prognosis of prostate cancer. Comparative proteomic technologies were used in the present study. Total protein from 10 cases of prostate cancer, benign prostatic hyperplasia and normal prostate tissue was extracted and separated by two-dimensional electrophoresis (2-DE). Proteins expressed differentially by more than 2-fold were selected for matrix-assisted laser desorption/ionization time-of-flight mass spectrometry (MS) and biological information analysis. The 2-DE patterns of the proteins from the normal prostate, benign prostatic hyperplasia and prostate cancer tissues were successfully identified. The average numbers of protein spots were 3,066, 3,289 and 2,986, respectively. There were 31 spots with a difference of more than 2-fold. A total of 18 proteins were identified by MS and database searches. Of these 18 proteins, the most significant differential expression was that of 90K/Mac-2BP. Functional analysis demonstrated that 90K/Mac-2BP (Mac-2 binding protein) overexpression is correlated with the occurrence, proliferation, differentiation and metastasis of cancer cells. The proteomic approach used in the present study was effective and is feasible for identifying differentially expressed proteins in prostate cancer, benign prostatic hyperplasia and normal prostate tissues. 90K/Mac-2BP may be important for the early diagnosis and prognosis of prostate cancer and may also be associated with the molecular mechanisms of prostate cancer development.

## Introduction

Prostate cancer has become the most common cancer among males in the United States, surpassing lung cancer, and is the second most common cause of cancer-related mortality (1,2). In China, the incidence of prostate cancer is gradually increasing. Due to the absence of a reliable early diagnostic method, most prostate cancer incidences are diagnosed during the late stages, leading to high mortality. As the elderly population increases, prostate cancer becomes a significant international health problem. Prostate cancer has a wide range of individual differences and variability and is more invasive than other tumors. No standard methods for prostate cancer prevention, early diagnosis, treatment or prognosis are currently available (3). Prognostic indicators that benefit the early diagnosis and treatment of prostate cancer are required to establish a reliable diagnostic method (4).

In the present study, the protein expression of prostate cancer, normal prostate and benign prostatic hyperplasia tissues were screened using two-dimensional gel electrophoresis (2-DE) and mass spectrometry (MS) analysis to identify early diagnostic, predictive, and prognostic markers for prostate cancer.

## Materials and methods

### Samples

All tissue samples for proteomic research were obtained from the Department of Urology, Guizhou Provincial People’s Hospital (Guiyang, China) between July 2009 and May 2001. The samples included 10 each of prostate cancer, benign prostatic hyperplasia and normal prostate tissues, respectively, The patients were 55–76 years of age, with a mean age of 65.8 years and a median age of 65 years. The patients did not receive preoperative radiotherapy or chemotherapy. All specimens were pathologically confirmed to be prostate cancer, benign prostatic hyperplasia or normal prostate tissue. All samples were immediately stored at −80°C. This study was conducted in accordance with the declaration of Helsinki. This study was conducted with approval from the Ethics Committee of the People’s Hospital of Wuhan University (Wuchang, China). Written informed consent was obtained from all participants.

### Tissue protein extraction

Up to 100 mg of tissue samples were collected and cut into sections. Cell lysate (∼300 ml) and phenylmethylsulfonylfluoride (200 mmol/l) were added to the samples, which were then homogenated, ultrasonically treated and centrifuged at 20,000 × g for 30 min at 4°C. The supernatant was then transferred into a new EP tube. The protein was purified according to the kit instructions (Goodtime Biological Technology Co., Ltd., Wuhan, China), and the protein concentration was measured.

### 2-DE gel electrophoresis

The protein sample was placed in an isoelectric focusing (IEF) strip slot, and dry-immobilized pH gradient strips were added for the first-dimension electrophoresis. The IEF parameters were set as follows: 30 V, 12 h (strip rehydration); 500 V, 1 h; 1 kV, 1 h; and 8 kV, 30 min. Following IEF, the balance strip was balanced in a buffer. The tape was transferred to 12.5% SDS-PAGE gel (GE company, USA) for the second electrophoresis. The parameters were set to 2 W/gel for 50 min, and then changed to 17 W/gel until the indicator reached the bottom edge of the electrophoresis gel. The gel was stained according to the instructions of the Deep Purple staining method.

### Image acquisition and analysis

The gel was analyzed using a Typhoon 9400 gel imaging system. The mass spectrum was analyzed using Image Master Software. The points of difference were found by gel image recognition and analysis. Statistical analysis was performed using a Student’s t-test. Protein spots expressed differentially by >2-fold were selected. P<0.05 was considered to indicate a statistically significant protein spot.

### Obtaining the mass spectra

The stained gel was placed in a Spothandling workstation. The protein spots were cut automatically before being digested with trypsin and extracted. Following desalting, the sample was analyzed on a target mass matrix-assisted laser desorption/ionization time-of-flight (MALDI TOF) mass spectrometer. Peptide mass fingerprinting (PMF) and the five highest ion peaks of peptide tandem MS (MS/MS) spectra were obtained.

### MS data analysis

The MS data were searched using the Mascot software in the NCBInr database. The parameters were *Homo sapiens* species, 75 ppm peptide tolerance and 0.2 ion fragment tolerance. A protein search score >95 was considered to indicate successful protein identification.

### Statistical analysis

Results are expressed as means ± standard deviation. The t-test comparison was used to conduct a cross-sectional study of all continuous data. The Pearson correlation coefficient was used for correlation analysis. P<0.05 was considered to indicate a statistically significant difference.

## Results

### Gel image analysis

**Figure 1:**
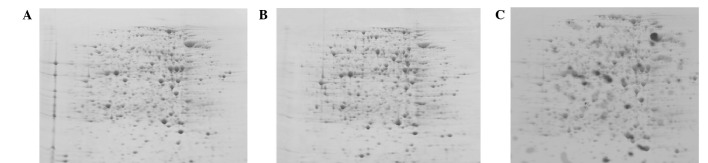
Gel image analysis results. (A) Normal group; (B) benign prostate hyperplasia group; (C) prostatic hyperplasia group.

The 2-D maps revealed different protein spots in the 3 groups. The number of protein spots in the prostate cancer and benign prostatic hyperplasia tissues was greater than those in normal tissues (Fig. 1).

### Map analysis

The average number of protein spots on the normal prostate, prostate cancer and benign prostatic hyperplasia tissue gel electrophoresis were 3,066, 3,289 and 2,986, respectively. One of each group of the gels was set as a reference, and the gel patterns for 8 spots were subjected to matching analysis. The average protein points in the prostate normal tissue gel electrophoresis was used as the reference gel, and then the average protein points of the gland carcinoma tissue and benign prostatic hyperplasia tissue were compared with these, the obtained values were 85.6% and 81.3%, respectively. Further repeatability analysis revealed that the 8 spots of protein patterns in prostate cancer and benign prostatic hyperplasia were highly repeatable. When the expression of protein spots and expression levels of all protein spots totalled >3 and the same protein changes occurred in the three gel maps, the protein spots were considered to be differential.

### MS results

From the 2-D maps of prostate cancer and benign prostatic hyperplasia, 31 protein spots were identified as being differentially expressed by >2-fold and were selected for MALDI-TOF-MS/MS analysis. A total of 31 PMFs were obtained in 31 points.

### Protein identification

The PMF data and a ProFound query were used to search the NCBInr database and protein points were identified by comparison and PMF of the proteins. A total of 18 matched scores equalled >63 points (Table I).

## Discussion

In prostate cancer diagnosis, the prostate-specific antigen (PSA) is a relative biological indicator, but it is not very specific for this type of cancer (5). Identifying the tumor markers of prostate cancer is extremely important for prostate cancer prevention, treatment and prognosis.

Oncogenes are genes that control cell growth and have the potential to induce cells to transform into malignant cells. The products of abnormal gene expression in cancer genes allow cells to proliferate indefinitely, allowing immortalization to occur. Searching for cancer genes and taking appropriate measures in cancer treatment are important means of tumor gene therapy. 90K/Mac-2BP (Mac-2 binding protein, cytomegalovirus-2 binding protein) was identified in 1986 by Iacobelli *et al* and is a tumor-associated antigen (6). It was detected in culture media using the monoclonal antibody SP-2 from CG-5 breast cancer cell lines and has 90-kDa subunits, hence the name 90K. 90K is a highly glycosylated secreted protein, a plant hemagglutinin Mac-2 (galectin-3) ligand. Studies have confirmed that 90K is a serum diagnostic marker for lymphoma (6) as well as lung (7), breast (8), liver (9) and ovarian cancer (10), among others (11,12). 90K is associated with the host response to tumors, which is capable of inducing the expression of cytokines, such as IL-1, IL-2 and IL-6 (13). 90K is also highly expressed in proteomic analysis of prostate cancer, indicating that it is strongly correlated with prostate cancer (14). The present study revealed that 90K is highly expressed in prostate adenocarcinoma and its expression is statistically significant in benign prostatic hyperplasia and normal prostate tissues.

Previous studies have indicated that 90K promotes tumor metastasis and that the tumor promotion mechanism in metastasis is associated with galectins. Galectins (galectin-1 and galectin-3) mainly mediate cell-cell and cell-matrix interactions and are involved in cell proliferation and angiogenesis. Galectins are also important in tumor invasion and metastasis. Endogenous galectin ligands include laminin, fibronectin, lysosome-associated membrane protein and 90K. Galectin-1 and galectin-3 are highly expressed in tumor tissues and galectin-3 confers high metastatic potential. Inohara *et al*(15) revealed that 90K cross-links adjacent cells expressing galectin-l and galectin-3 residues, mediating the blood flow in tumor cell aggregation and thrombus formation and thus promoting swelling and metastatic spread. 90K-bound galectins use a combination of sugar chains. Glycosylation inhibitors inhibit 90K and galectin cross-linking (16). 90K also promotes matrilysin factor expression (17). 90K and galectins are located in the extracellular matrix and various matrix components mediate cell adhesion. Therefore, 90K and galectins enhance tumor cell adhesion and the extracellular matrix to promote the formation of new tumor cell clones. The adhesion of the extracellular matrix may aid tumor cells avoid apoptosis.

To conclude, the proteomic approach used in the present study was effective and is feasible for identifying differentially expressed proteins in prostate cancer, benign prostatic hyperplasia and normal prostate tissues. 90K/Mac-2BP may be important for the early diagnosis and prognosis of prostate cancer and may also be associated with the molecular mechanisms of prostate cancer development.

## Figures and Tables

**Table I:** Protein points identified by comparison and peptide mass fingerprinting of the proteins.

Protein No.	Accession	Protein name	Molecular weight	PI	Peptide identified	Score
1	L13210	Human Mac-2 binding protein	92,000	8.45	79	247
2	XP_001352096	Phosphoglycerate kinase	45,569	7.63	15	110
3	AAV38387	Adenylate kinase 1	21,735	8.73	11	108
4	CAB45236	Catalase	59,947	6.90	17	105
5	C3HU	Complement C3 precursor	188,585	6.02	23^a^	95
6	1K1K_B	Carbonmonoxyhemoglobin C	15,970	7.98	12^a^	88
7	BAB17688	Heat shock protein hsp70 homologue Pfhsp70-3	71,945	5.90	30^a^	84
8	AAD29608	kappa 1 immunoglobulin light chain	26,181	5.72	12^a^	78
9	XP_001350775	Calcyclin binding protein, putative	30,547	8.36	10	73
10	P02735	Serum amyloid A protein	13,581	6.28	75	112
11	Q03591	Factor H-related protein 1	38777	7.75	37	86
12	Q9H7N9	FLJ00029 protein	27,133	8.95	20	64
13	O75635	Serpin B7	43,166	6.34	31	57
14	O43866	CD5 antigen-like	39,603	5.28	69	212
15	P05452	Tetranectin	22,951	5.52	43	63
16	P00739	Haptoglobin-related protein	39,496	6.42	24	84
17	P02766	Transthyretin	15,991	5.52	57	128
18	O75636	Ficolin-3	33,395	6.20	38	107

a Phosphorylated peptides identified.
